# Care-seeking correlates of acute respiratory illness among sheltered adults experiencing homelessness in Seattle, WA, 2019: a community-based cross-sectional study

**DOI:** 10.3389/fpubh.2023.1090148

**Published:** 2023-06-20

**Authors:** Julia H. Rogers, Stephen E. Hawes, Caitlin R. Wolf, James P. Hughes, Janet A. Englund, Lea M. Starita, Helen Y. Chu

**Affiliations:** ^1^Division of Allergy and Infectious Diseases, Department of Medicine, University of Washington, Seattle, WA, United States; ^2^Department of Epidemiology, University of Washington, Seattle, WA, United States; ^3^Vaccine and Infectious Disease Division, Fred Hutchinson Cancer Research Center, Seattle, WA, United States; ^4^Department of Biostatistics, University of Washington, Seattle, WA, United States; ^5^Division of Pediatric Infectious Diseases, Department of Pediatrics, Seattle Children’s Research Institute, University of Washington, Seattle, WA, United States; ^6^Department of Genome Sciences, University of Washington, Seattle, WA, United States

**Keywords:** health care utilization, respiratory virus infection, homeless shelters, cross-sectional study, predictors of interest

## Abstract

**Objective:**

Multifarious barriers to accessing healthcare services among people experiencing homelessness (PEH) lead to delays in seeking care for acute infections, including those caused by respiratory viruses. PEH are at high risk of acute respiratory illness (ARI)-related complications, especially in shelter settings that may facilitate virus spread, yet data characterizing healthcare utilization for ARI episodes among sheltered PEH remained limited.

**Methods:**

We conducted a cross-sectional study of viral respiratory infection among adult residents at two homeless shelters in Seattle, Washington between January and May 2019. We assessed factors associated with seeking medical care for ARI via self-report. We collected illness questionnaires and nasal swabs were tested for respiratory viruses by reverse transcription quantitative real-time PCR (RT-qPCR).

**Results:**

We observed 825 encounters from 649 unique participants; 241 (29.2%) encounters reported seeking healthcare for their ARI episode. Seasonal influenza vaccine receipt (adjusted prevalence ratio [aPR] 1.39, 95% CI 1.02–1.88), having health insurance (aPR 2.77, 95% CI 1.27–6.02), chronic lung conditions (aPR 1.55, 95% CI 1.12-2.15), and experiencing influenza-like-illness symptoms (aPR 1.63, 95% CI 1.20 - 2.20) were associated with increased likelihood of seeking care. Smoking (aPR 0.65, 95% CI 0.45-0.92) was associated with decreased likelihood of seeking care.

**Discussion:**

Findings suggest that care seeking for viral respiratory illness among PEH may be supported by prior engagement with primary healthcare services. Strategies to increase healthcare utilization may lead to earlier detection of respiratory viruses.

## 1. Background and significance

Respiratory pathogens are the leading infectious cause of death in the United States (U.S.) and pose a disproportionately high risk to people experiencing homelessness (PEH) due to shelter crowding and poor ventilation (1–5). Past studies have indicated that pneumonia infection rates in the homeless are substantially higher than in the general population, and that mortality in the homeless due to respiratory infections is about seven times greater (6, 7).

For PEH, healthcare can compete with more immediate needs, such as obtaining food and housing (8). Studies have suggested that frequent utilization of emergency health services observed among PEH may be the result of their disproportionately high rates of chronic and acute health conditions, as well as serve as an indicator of poor access to primary healthcare in ambulatory settings (9). However, research has shown that those experiencing homelessness are likely to delay seeking care for acute infections, and when they do they are largely dependent on hospital and emergency services (10, 11). Factors such as health insurance status, duration of homelessness, and stability of shelter arrangements have been examined in relation to primary care access for any aggregated acute or chronic conditions within this population, as well as mental health and substance abuse services (12–16). The frequency of healthcare utilization among homeless populations, specifically for acute respiratory illness (ARI) episodes or influenza-like-illness (ILI), has not been assessed in prior studies. Understanding when and how sheltered PEH seek care for ARI is critical to mitigating the rapid spread of respiratory viruses during both seasonal and pandemic outbreaks (17).

The objective of this study was to evaluate viral respiratory infection and seeking healthcare in sheltered adult PEH, and assess factors that may impact the decision to seek healthcare for ARI in a community-based setting.

## 2. Methods

### 2.1. Study setting

Between January 21, 2019 and May 16, 2019, we conducted prospective surveillance of adult shelter residents aged 18 years and older experiencing homelessness who identified their primary residence as one of two shelters in Seattle, WA. Participants were enrolled through the Seattle Flu Study (SFS). Briefly, the SFS aimed to gather data from community and hospital sites to advance the understanding of how respiratory pathogens enter and spread in a population, create a city-wide platform for testing novel interventions that may limit or contain outbreaks, and establish a deployable infrastructure for future pandemics (18). Data collected for this analysis were from an initial pilot period to assess feasibility and acceptability of standard respiratory virus surveillance in a shelter setting.

### 2.2. Study design

We conducted a prospective cross-sectional study examining predictors of having sought healthcare among adult shelter residents with ARI. The primary exposure in the analysis is a respiratory illness with detection of at least one viral pathogen, referred to as a viral ARI (binary yes/no). The primary outcome of this study was study encounters where the study participant self-reported having sought clinical care in the past week for their current ARI episode from a medical provider (binary yes/no). We explored various clinical and demographic exposures as correlates to address our primary aim (see Supplementary material for more details).

### 2.3. Study subjects

Shelter residents were eligible to participate in the study if they reported at least two symptoms of ARI in the last 7 days. These symptoms included feeling feverish; runny or stuffy nose; headache; rash; cough; myalgia; excess fatigue; diarrhea; sore throat; increased trouble with breathing; nausea or vomiting; and ear pain or ear discharge.

Potential participants were screened and enrolled in-person by trained research assistants at one of two shelters (A and B) 2–4 days per week. Shelter A provided services to all adults 18 years and above with a 200-bed capacity, while Shelter B limited services to men aged 50 years and above and had a 212-bed capacity. New shelter client intake varies by time and location, but based on census data provided by shelter management from 2021, each shelter processes an average of 30–35 new residents each month. Staff at kiosks provided promotional and informational materials. Participants were recruited using convenience sampling methods.

### 2.4. Data collection

For those that met the eligibility criteria, we obtained informed consent and collected demographic, clinical, and behavioral data via an app-based questionnaire (Audere, Seattle, Washington, United States) on an electronic tablet. All data collected and generated was housed by Audere, a centralized electronic data capture system. Participants were given the choice of completing the questionnaire themselves on the tablet or having the study staff read it aloud and record their responses. Reasons participants may not have been capable of completing the questionnaire alone included if they did not have the appropriate corrective aids (i.e., glasses, contacts) for poor vision; were unfamiliar/uncomfortable with the tablet technology; or possessed a low English-language reading level. A Spanish-language version of the questionnaire was also available. Research assistants collected mid-nasal samples from participants using a sterile nylon flocked nasal swab (Quidel, San Diego, CA) and immediately placed in universal transport media.

### 2.5. Specimen processing and laboratory testing

Samples were transported to the University of Washington Brotman Baty Institute for Precision Medicine and the Northwest Genomics Center in universal transport media (Becton, Dickinson and Company, Franklin Lakes, NJ) at 4°C for up to 7 days prior to aliquoting and then stored at −80°C until testing. Samples were purified for total nucleic acids using the Roche MagnaPure 96 DNA and viral NA small volume kit, Viral NA Universal SV 4.0 protocol (200 μL input, 50 μL elution). Extracted nucleic acids were screened for the presence of 27 respiratory pathogens by TaqMan reverse transcription-qPCR (RT-qPCR) on the OpenArray platform with a custom array (Thermo Fisher) (19). Respiratory pathogens tested for included strains of the following viruses: influenza; respiratory syncytial virus (RSV); human parainfluenza (hPIV); human coronavirus (HCoV) sub-types HCoV-229E, HCoV-OC43, HCoV-NL63 and HCoV-HKU1; human metapneumovirus (hMPV); rhinovirus (RV); mumps; measles; human parechovirus; enterovirus; human bocavirus; and adenovirus (AdV). In addition to the viral pathogens, we also tested samples for *Bordetella pertussis*, *Streptococcus pneumoniae* (*S. pneumoniae*), *Mycoplasma pneumoniae* (*M. pneumoniae*), and *Chlamydia pneumoniae* (*C. pneumoniae*). These pathogens were not analyzed as primary exposures.

### 2.6. Statistical analysis

We performed descriptive statistics of sociodemographic variables per unique participant and clinical/laboratory and health access variables per study encounter, by whether clinical care was sought, to address our first primary aim. Variables analyzed include self-reported age, sex, race, duration of homelessness, smoking status, employment status, number of days since symptom onset, chronic comorbidities, ILI status, and *S. pneumoniae* carriage. Access to healthcare variables included health insurance status and influenza vaccination status. Any enrollments from the same unique participant that were < 14 days apart were excluded from analysis.

To determine correlates of seeking healthcare given an ARI illness episode, simple logistic regression was first performed. We then evaluated the relationship of each covariate with healthcare seeking after adjusting for the other variables. Covariates assessed included all variables listed in Tables 1, 2. Covariates were retained in the model if their inclusion changed the Prevalence Ratio (PR) estimate by ≥10%. This was determined for each variable independently as opposed to automatically. A generalized estimating equation (GEE) model for each correlate of interest was constructed to account for repeated observations of individuals within a specific shelter. Adjusted PR’s (aPR) with 95% confidence interval (CI) were estimated and any intervals that did not contain the null hypothesis value of 1 were considered statistically significant.

**Table 1:** Baseline sociodemographic characteristics of unique participants upon first acute respiratory illness (ARI) episode among sheltered homeless individuals, Seattle Flu Study January 2019—May 2019 (*N* = 649).

	Did not seek healthcare *N* = 468 (%)	Sought healthcare *N* = 181 (%)	Total *N* = 649 (%)
Sociodemographic			
Age			
Mean (SD)	52.8 (11.0)	53.6 (11.9)	53.1 (11.3)
Median [Min, Max]	55.0 [20, 78]	56.0 [24, 88]	55.0 [20.0, 88.0]
Male sex	369 (78.8)	127 (70.2)	496 (76.4)
Race/Ethnicity*			
White	207 (44.2)	79 (43.6)	286 (44.1)
Black or African American	149 (31.8)	56 (30.9)	205 (31.6)
Asian	13 (2.8)	5 (2.8)	18 (2.8)
Native Hawaiian or Pacific Islander	3 (0.6)	1 (0.6)	4 (0.6)
American Indian or Alaskan Native	5 (1.1)	3 (1.7)	8 (1.2)
Other	45 (9.6)	13 (7.2)	58 (8.9)
Multiple races	33 (7.1)	18 (9.9)	51 (7.9)
Missing	13 (2.8)	6 (3.3)	19 (2.9)
Hispanic/Latinx			
Yes	44 (9.4)	14 (7.7)	58 (8.9)
No	419 (89.5)	164 (90.6)	583 (89.8)
Missing	5 (1.1)	3 (1.7)	8 (1.2)
Duration of homelessness			
≤6 months	101 (21.6)	38 (21.0)	139 (21.4)
7–12 months	68 (14.5)	27 (14.9)	95 (14.6)
13–24 months	58 (12.4)	25 (13.8)	83 (12.8)
>24 months	225 (48.1)	85 (47.0)	310 (47.8)
Missing	16 (3.4)	6 (3.3)	22 (3.4)
Smoker^†^			
Yes	379 (81.0)	129 (71.3)	508 (78.3)
No	88 (18.8)	51 (28.2)	139 (21.4)
Missing	1 (0.2)	1 (0.6)	2 (0.3)
Number of encounters per unique individual^‡^			
1	386 (78.9)	143 (69.1)	529 (81.5)
2	72 (14.7)	42 (20.3)	84 (12.9)
≥3	31 (6.3)	22 (10.6)	36 (5.5)
Shelter site			
A	234 (50.0)	113 (62.4)	347 (53.5)
B	234 (50.0)	68 (37.6)	302 (46.5)
Chronic comorbidity^§^			
Yes	181 (38.8)	73 (40.3)	254 (39.1)
No	287 (61.2)	108 (59.7)	395 (60.9)

*Percentages may not add up to 100 as categories are not mutually exclusive.

^†^Tobacco, marijuana, or vaping.

^‡^Columns may not add up to total N as categories are not mutually exclusive due to differential care seeking behavior over multiple ARI episodes.

^§^Includes chronic obstructive pulmonary disorder/emphysema, chronic bronchitis, asthma, diabetes, or cancer.

**Table 2:** Self-reported characteristics of all acute respiratory illness (ARI) episodes among shelter residents, January 2019—May 2019 (*N* = 825).

	Did not seek healthcare *N* = 584 (%)	Sought healthcare *N* = 241 (%)	Total *N* = 825 (%)
Clinical and laboratory			
Viral ARI			
Yes	110 (18.8%)	44 (18.3%)	154 (18.7%)
No	474 (81.2%)	197 (81.7%)	671 (81.3%)
Coinfection (≥2 viruses detected)			
Yes	7 (1.20%)	4 (1.66%)	11 (1.33%)
No	577 (98.8%)	237 (98.3%)	814 (98.7%)
Symptom duration			
1–2 days	76 (13.0%)	25 (10.4%)	101 (12.2%)
3–6 days	143 (24.5%)	38 (15.8%)	181 (21.9%)
≥7 days	364 (62.3%)	178 (73.9%)	542 (65.7%)
Missing	1 (0.2%)	0 (0%)	1 (0.1%)
Influenza-like-illness*			
Yes	235 (40.2%)	126 (52.3%)	361 (43.8%)
No	349 (59.8%)	115 (47.7%)	464 (56.2%)
*S. pneumoniae* carriage			
Yes	128 (21.9%)	58 (24.1%)	186 (22.5%)
No	456 (78.1%)	183 (75.9%)	639 (77.5%)
Access to care			
Health insurance^†^			
Yes	503 (86.1%)	223 (92.5%)	726 (88.0%)
No	56 (9.6%)	8 (3.3%)	64 (7.8%)
Missing	25 (4.3%)	10 (4.1%)	35 (4.2%)
Influenza vaccine received within the last 12 months			
Yes	254 (43.5%)	123 (51.0%)	377 (45.7%)
No	326 (55.8%)	113 (46.9%)	439 (53.2%)
Missing	4 (0.7%)	5 (2.1%)	9 (1.1%)
Received treatment from a medical provider^‡^			
Yes	31 (5.3%)	120 (49.8%)	151 (18.3%)
No	551 (94.3%)	119 (49.4%)	670 (81.2%)
Missing	2 (0.3%)	2 (0.8%)	4 (0.5%)

*Self-reported fever and cough, or fever and sore throat.

^†^Self-reported coverage by government, private, or other.

^‡^Antiviral or antibiotics.

We defined the prevalence of viral ARI detection as the number of episodes with at least one viral pathogen tested positive out of the total number of illness episodes. Separate GEE models were fit to assess whether seeking healthcare varied depending on whether a specific virus detected. Symptom duration and having received treatment for an illness episode were selected *a priori* as confounders for these models.

A complete case analysis was performed as no variables had missingness that exceeded 10% of observations. As a sensitivity analysis, type of self-reported chronic comorbidity was examined to discern whether certain conditions had a stronger association with the outcome of healthcare utilization. Statistical analyses were performed using R Statistical Software (Version 3.6.0. “Planting of a Tree,” Foundation for Statistical Computing, Vienna, Austria).

This study was approved by the Human Subjects Division of the University of Washington Institutional Review Board (STUDY00006181) and was prepared using the Strengthening the Reporting of Observational Studies in Epidemiology (STROBE) reporting guidelines.

## 3. Results

Overall, 649 unique participants had 825 illness episodes, or study encounters, across the two sites from January 21, 2019 through May 16, 2019. There were 529 (81.5%) participants with one encounter, 84 (12.9%) with two, and 36 (5.5%) with three or more. Of the 120 participants with multiple encounters, 47 had differential care seeking behavior over multiple ARI episodes. Unique participants’ sociodemographic characteristics, stratified by care seeking at first ARI episode, are shown in Table 1. The mean age of participants was 53 years (SD: 11), and 496 (76.4%) were male. White (44.1%) and Black or African American (31.6%) were the predominant racial groups represented. The majority were smokers (78.3%), and nearly half (47.8%) reported having experienced homelessness for more than 2 years. A total of 254 (39.1%) participants had at least one chronic comorbidity. Each shelter represented approximately half of our study’s unique participants.

Among all ARI episodes (*N* = 825), 241 (29.2%) were from participants that reported having sought healthcare prior to that study encounter (Table 2). A majority (*n* = 726, 88%) were from participants with health insurance at the time of the illness episode; 151 (18.3%) indicated having received antiviral or antibiotic treatment from a medical provider for their ARI episode. There were 154 (18.7%) encounters where the participant tested positive for at least one respiratory virus.

A marginally larger proportion of ARI episodes with symptom onset occurring ≥7 days ago had sought healthcare for their symptoms (62.3% vs. 73.9%). Of the 241 episodes where healthcare was sought, 126 (52.3%) had ILI symptoms. More than half (51%) of episodes from participants that sought healthcare reported having received an influenza vaccine within the last 12 months, and 92.5% of episodes where healthcare was sought were experienced by participants with health insurance. Of the ARI episodes where the participant sought healthcare, 120 (49.8%) included receiving treatment from a clinician.

In adjusted analyses, a number of predictive factors were found to be significantly associated with seeking healthcare for an ARI episode (Table 3). Individuals identifying as American Indian or Alaskan Native (AIAN) were associated with a 38% higher prevalence than those that identified as White (aPR 1.38, 95% CI 1.06–1.79). Smokers were 35% less likely to have sought healthcare for their illness (aPR 0.65, 95% CI 0.45–0.92). Those with chronic lung conditions were 55% more likely to have sought healthcare (aPR 1.55, 95% CI 1.12–2.15). However, neither having been diagnosed with cancer nor diabetes was significantly associated with this outcome (aPR 0.64, 95% CI 0.31–1.33, and aPR 1.43, 95% CI 0.92–2.20, respectively). Those that reported experiencing ILI symptoms were 63% more likely to have sought healthcare (aPR 1.63, 95% CI 1.20–2.20) when compared to those with other symptom profiles. Those with health insurance were nearly three times as likely to have sought healthcare when compared to uninsured participants (aPR 2.77, CI 95% 1.27–6.02). Those that received the influenza vaccine within the last 12 months were 39% more likely to have sought healthcare when compared to those who were not vaccinated (aPR 1.39, 95% CI 1.02–1.88).

**Table 3:** Correlates of having sought healthcare for an acute respiratory illness (ARI) episode in the past 7 days among sheltered homeless individuals, Seattle Flu Study, January 2019—May 2019 (*n* = 825).

	Sought healthcare (%)	Adjusted estimates aPR (95% CI)
Sociodemographic		
Age*		
18–49	73 (31.5)	Reference
50–64	132 (26.5)	1.06 (0.72–1.55)
≥65	36 (37.9)	1.49 (0.90–2.47)
Male sex (ref. female)*	167 (26.6)	0.77 (0.52–1.15)
Race/Ethnicity^†^		
White	111 (29.8)	Reference
American Indian or Alaskan Native	6 (42.9)	**1.38 (1.06–1.79)**
Asian	5 (25.0)	0.97 (0.74–1.26)
Black or African American	68 (27.8)	1.02 (0.78–1.32)
Native Hawaiian or other Pacific Islander	2 (33.3)	1.29 (0.99–1.68)
Multiple races	26 (35.1)	1.26 (0.97–1.63)
Hispanic or Latinx (ref. no)^‡^	19 (27.1)	0.80 (0.45–1.41)
Duration of homelessness		
≤6 months	57 (30.0)	Reference
7–12 months	32 (29.1)	0.95 (0.56–1.58)
13–24 months	34 (30.6)	1.01 (0.61–1.71)
>24 months	111 (28.8)	0.93 (0.56–1.56)
Smoking (ref. no)		**0.65 (0.45–0.92)**
Clinical and laboratory		
Viral ARI detected		
No	197 (29.4)	Reference
Yes	44 (28.6)	0.96 (0.65–1.42)
Coinfection (≥2 viruses detected)^§^	4 (36.4)	1.21 (0.35–4.26)
Symptom duration		
1–2 days	25 (24.8)	Reference
3–6 days	38 (21.0)	0.81 (0.46–1.45)
≥7 days	178 (32.8)	1.49 (0.84–2.65)
Chronic lung condition (ref no)	85 (36.2)	**1.55 (1.12–2.15)**
Cancer (ref no)^|^	12 (24.5)	0.64 (0.31–1.33)
Diabetes (ref no)	37 (35.9)	1.43 (0.92–2.20)
Influenza-like-illness	126 (34.9)	**1.63 (1.20–2.20)**
*S. pneumoniae* carriage	58 (31.2)	1.11 (0.78–1.59)
Access to care		
Health insurance^¶^	223 (30.7)	**2.77 (1.27–6.02)**
Influenza vaccine received within the last 12 months	123 (32.6)	**1.39 (1.02–1.88)**

*Adjusted for shelter site identifier.

^†^Adjusted for sex.

^‡^Adjusted for smoking status.

^§^Adjusted for influenza-like-illness.

^|^Adjusted for age category.

^¶^Adjusted for race/ethnicity.

**Figure 1:**
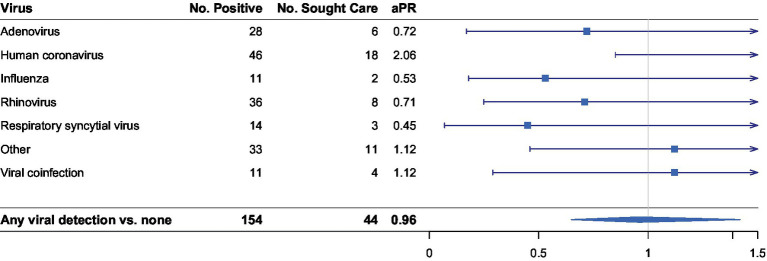
Stratified analysis of association between virus detected and having sought healthcare with at least one viral respiratory pathogen detected (*n* = 154). Number of positives per pathogen do not add up to 154 as positive detection was not mutually exclusive. Adjusted prevalence ratios (aPR) are displayed on the logarithmic scale. Human coronavirus (HCoV) sub-types include HCoV-229E, HCoV-OC43, HCoV-NL63, and HCoV-HKU1. “Other” viral pathogens detected include enterovirus, human metapneumovirus, and human parainfluenza viruses. All regression models were adjusted for number of days since symptom onset and having received treatment for an illness episode.

We assessed encounter-specific healthcare seeking by viral infection. Comparisons are first for each specific pathogen versus not having that pathogen, and then for any identified pathogen versus no pathogen. Of the 825 ARI episodes, 154 (18.6%) were positive for at least one of the 27 viral pathogens tested for, and we observed 11 episodes of viral coinfection. Overall, no statistically significant difference was detected when comparing a viral-positive ARI episode and a viral-negative episode (aPR 0.96, 95% CI 0.65–1.42, Figure 1). There was also no significant difference detected between participants with a certain virus and seeking healthcare, including HCoV, influenza, and RSV.

## 4. Discussion

We found that less than a third of ARI episodes reported by participants had sought healthcare for their symptoms in the past week from a medical professional. AIAN race, smoking, having a chronic lung condition, self-assessed ILI symptoms, health insurance status, and seasonal influenza vaccination status were significantly correlated with having sought healthcare. Participants that tested positive for a specific virus were not more likely to seek care compared to other viral-positive ARI episodes observed.

The reported proportion of ARI episodes that resulted in seeking care from a clinical provider is difficult to contextualize as data concerning healthcare utilization for ARI in this population is scant. Past national-level cohort studies conducted not in the context of a viral respiratory pandemic (e.g., H1N1pdm09, COVID-19) have shown that the majority of individuals with ARI did not access healthcare, indicating that care-seeking in the general population is not common (20).

Factors indicative of a shelter participant’s access to care were particularly strongly associated with having sought clinical care for their illness. We found health insurance coverage to be the strongest predictor of having sought healthcare for ARI. This is concordant with research assessing use of ambulatory care for any reason, and factors associated with decreased barriers to care in homeless populations (12). Seattle’s 2018 One Night Count Survey, which reported 52% of respondents as unsheltered, found 19% of respondents were insured by Medicaid/Medicare (21). This study observed 87.4% insurance coverage, comparable to coverage in homeless populations of other US-metropolitan areas in recent years since the advent of the Affordable Care Act (22, 23). Despite high rates of insurance coverage, however, homeless populations are observed to be additionally hindered and/or driven in their healthcare use by barriers directly related to the organization of care (24, 25).

The significant association detected between seasonal influenza vaccination and seeking care is also consistent with findings from other studies (13). In comparison, this relationship has not been reported as significant in the general population (20). Overall influenza vaccine coverage reported here is higher than previously reported rates observed in homeless populations both inside and outside of the US (25–36%) (3, 26). It is possible that having a chronic illness may have modified the correlation between influenza vaccine uptake and having sought healthcare for an ARI if vaccination due to their high risk status is being encouraged by these patients’ healthcare providers.

The association observed between having a chronic lung condition and seeking care is consistent with findings from studies assessing predictors of healthcare utilization in other populations (20, 27). Participants with chronic lung conditions were specifically assessed rather than having any chronic comorbidity as these conditions were disproportionately reported by participants in our study when compared to the general adult population (39% vs. 15%) (28). These individuals may be at risk of disease-related exacerbations commonly associated with viral respiratory illness (29, 30). Similar rates of care seeking among the general population with these conditions suggests that they are more cognizant of their high risk condition due to provider health education and/or having pre-existing linkage to care, compared to homeless individuals without a chronic lung condition. The inverse association we detected between being a smoker and seeking care, however, is concerning as cigarette smoking use has been shown to increase the incidence, duration and severity of viral respiratory infections (31).

Fulfillment of the ILI definition during an ARI episode was found to be independently associated with seeking care in this study. Prior research using syndromic surveillance of national-level populations have reported inconsistent findings assessing ILI as a predictive factor (20, 32, 33). One such study that observed an increased likelihood of visiting a health facility when participants self-diagnosed their illness as influenza compared to cold, and suggested that individuals are capable of gaging their disease severity and categorizing more severe episodes as “flu-like” (20). Another found between 40.9 and 46.8% of adult participants reporting ILI symptoms sought medical care, substantially higher when compared to the 33.3% in our study (34). Our significant findings of ILI as a predictor indicates that sheltered homeless populations’ care seeking behavior is impacted by specific symptom profiles, and that they are capable of disease severity self-assessment when symptoms are flu-like. These findings, however, may not be applicable to viral respiratory pandemic contexts, as evidenced by the increased availability of community-based testing and high proportion of asymptomatic SARS-CoV-2 infections observed throughout the COVID-19 pandemic (35, 36).

The lack of any significant association identified between viral presence and seeking care after stratifying by specific pathogen was unexpected. Among respiratory viruses tested for, influenza has the greatest overall impact with regards to both morbidity and mortality. This is more pronounced among older adults and those with underlying conditions such as chronic obstructive pulmonary disease (COPD), congestive cardiac failure, and diabetes (37), conditions that are well-represented in our study participants. Other studies have observed greater risk of complications and poorer outcomes for RSV and influenza in homeless patients (38). This is also at odds with our finding that those with ILI symptoms were more likely to have sought healthcare. These discordant findings may be due to this study having been conducted in a community-setting rather than clinical or hospital setting where severity estimates may be inflated due to selection bias.

The higher prevalence of seeking care given detection of common HCoV strains is notable, despite not being significant, as HCoV symptoms are typically mild. This observation may be related to longer acute symptom duration and viral shedding periods of HCoV strains when compared to influenza and other viral pathogens, but much about the epidemiology of HCoV is still unknown and additional exploration is required (39–41). The overall lack of associations and wide confidence intervals detected post-stratification may be due to low prevalence of many pathogens, as a small sample size may have hampered precise effect estimates. The overall finding that those with a viral-positive ARI episode were not more likely to have sought healthcare for treatment may indicate that healthcare utilization is not sought until later in the illness episode following the onset of severe or prolonged symptoms. This is supported by the higher prevalence of seeking care in those reporting ≥7 days of symptom duration observed in this study. In the context of the COVID-19 pandemic, this may have major implications for the demand of hospital resources if cases are not detected and clinically managed early on due to delayed access to testing in this population (42).

### 4.1. Limitations

Convenience sampling is a core limitation to this study. Voluntary participation through passive recruitment at shelters may have introduced selection bias and resulted in a spuriously low detection of viral positivity. Misclassification of self-assessed symptoms as criteria for enrollment is another concern. Given the high rates of underlying conditions in this population, associated chronic symptoms may have allowed individuals to enroll who did not have new acute respiratory illnesses. Sample collection from participants with ≥7 days of symptom duration may have failed to detect viral-positive ARI episodes if viral shedding was no longer occurring. True prevalence estimates of viral positivity may have been improved if screening for participation was based upon *new or worsening* symptoms only within the past 7 days (43). However, there was no significant difference when we conducted a sensitivity analysis to determine whether viral detection varied by the reported time since symptom onset. While enrollments not beginning until January of the 2018–2019 influenza season may have resulted in an under-detection of some viruses, surveillance from the Washington State Department of Health reported a peak in influenza cases in March 2019 (44, 45).

Temporality is another limitation in this study. The cross-sectional nature of the data means the temporal relationship and therefore causal inferences about predictive factors and having sought healthcare cannot be ascertained. Collecting samples at time of healthcare seeking decision making or collecting follow-up data in subsequent weeks to determine whether they sought healthcare before their illness episodes resolved, would have addressed this issue. This is recommended for future community-based studies of ARI healthcare seeking behavior but may prove difficult in transient homeless populations.

Potential unmeasured confounding variables include whether participants had a primary care provider and symptom severity. Distinction between type of healthcare provider participants saw for their ARI episode (e.g., emergency department, clinic, shelter nurse, etc.) was also not measured. Unmeasured behavioral health disorders or mental illnesses also may have played a confounding role in our analyses. Prior research has shown concurrent disorders of mental health diagnosis and problematic substance use to be associated with higher rates of healthcare utilization among homeless individuals and vulnerably housed adults (46). Based on published literature, we also expect respondent recall bias, as vaccination status and other clinical variables were by self-report (47, 48).

## 5. Conclusion

This study provides novel insights into predictive factors associated with sheltered PEH seeking care for ARI episodes. The relatively small proportion of symptomatic participants who sought healthcare, despite high levels of health insurance coverage, suggests challenges to the early identification of viral illness in this population if prior engagement has not been made with primary healthcare services. Our findings suggest that standard viral respiratory surveillance strategies that rely heavily on care-seeking individuals tested in a clinical setting are failing to detect a large proportion of infections among PEH.

Given the high vulnerability of sheltered PEH and their inability to adhere to physical distancing measures in a congregate setting, understanding when and how they seek care for ARI is critical to mitigating the rapid spread of respiratory viruses during both seasonal and pandemic outbreaks (17). As the COVID-19 pandemic continues to evolve, further research elucidating testing barriers for early detection of viral ARI episodes and improving linkage-to-care with health services before viral shedding peaks in shelter settings is vital.

## Data availability statement

The datasets generated for this study are available on request to the corresponding author.

## Ethics statement

The studies involving human participants were reviewed and approved by the University of Washington Institutional Review Board. The patients/participants provided their written informed consent to participate in this study.

## Author contributions

JR, SH, JH, CW, JE, LS, and HC conceived the study. JR, CW, HC, LS, and JE designed the study tools for data collection and analysis. JR, JH, JE, LS, and HC collected the data and supervised the data collection. JR, SH, JH, and HC analyzed the data and supervised the analysis. JR and HC wrote the manuscript. JR, SH, CW, JH, JE, LS, and HC edited the manuscript. All authors contributed to the article and approved the submitted version.

## Funding

This study was supported by donations from Gates Ventures. The funder did not have a role in study design, data collection, data analysis, interpretation, writing of the report or decision to submit.

## Conflict of interest

HC reported consulting with Ellume, Pfizer, the Bill and Melinda Gates Foundation, Glaxo Smith Kline, and Merck. She has received research funding from Gates Ventures, Sanofi Pasteur, and support and reagents from Ellume and Cepheid outside of the submitted work. JE reported research support from Merck, AstraZenecxa, Pfizer, and GlaxoSmithKline. She was a consultant for Meissa Vaccines, Sanofi Pasteur, and Astra Zeneca.

The remaining authors declare that the research was conducted in the absence of any commercial or financial relationships that could be construed as a potential conflict of interest.

## Publisher’s note

All claims expressed in this article are solely those of the authors and do not necessarily represent those of their affiliated organizations, or those of the publisher, the editors and the reviewers. Any product that may be evaluated in this article, or claim that may be made by its manufacturer, is not guaranteed or endorsed by the publisher.
