# Supplementary material for: Care-seeking correlates of acute respiratory illness among sheltered adults experiencing homelessness in Seattle, WA, 2019: a community-based cross-sectional study
Source: Front Public Health. 2023 Jun 20;11:1090148. doi: 10.3389/fpubh.2023.1090148 (PMC10319010; doi:10.3389/fpubh.2023.1090148)
Supplement: Supplementary file 1 [file Table_1.docx]

**Supplemental Materials**

*Questionnaire Variables*

Health insurance coverage included both private and public insurance plans. Smoking status was determined by asking participants if they smoked tobacco, marijuana or vaped. Alcohol consumption was assessed as a binary yes/no in response to the question “*Do you ever drink alcohol?”* Drug use was determined by asking whether a participated used any recreational drugs besides marijuana. Sex was self-reported by participants in response to the question “*What was your assigned sex at birth?”* ILI was defined as self-report of fever with cough or sore throat. A chronic condition was defined as presence of self-reported diabetes, cancer, chronic obstructive pulmonary disease (COPD), asthma, or bronchitis. A chronic respiratory condition was defined as reported COPD, asthma, or bronchitis. Influenza vaccination status was documented based on self-report of month-year of receipt in the last 12 months.
